# Recovery of right ventricular function and strain in patients with ST-segment elevation myocardial infarction and concurrent chronic total occlusion

**DOI:** 10.1007/s10554-021-02423-9

**Published:** 2021-09-23

**Authors:** Anna van Veelen, Joëlle Elias, Ivo M. van Dongen, Loes P. C. Hoebers, Bimmer E. P. M. Claessen, Truls Ramunddal, Peep Laanmets, Erlend Eriksen, René J. van der Schaaf, Robin Nijveldt, Jose P. S. Henriques, Alexander Hirsch

**Affiliations:** 1 Department of Cardiology, Heart Center, Amsterdam UMC, University of Amsterdam, Amsterdam Cardiovascular Sciences, Amsterdam, The Netherlands; 2 Department of Cardiology, Sahlgrenska University Hospital, Gothenburg, Sweden; 3 Department of Cardiology, North-Estonia Medical Centre, Tallinn, Estonia; 4 Department of Cardiology, Haukeland University Hospital, Bergen, Norway; 5 Department of Cardiology, OLVG Hospital, Amsterdam, The Netherlands; 6 Department of Cardiology, Radboud University Medical Center, Nijmegen, The Netherlands; 7 Department of Cardiology, Erasmus Medical Center, University Medical Center Rotterdam, Room Rg-419, Dr. Molewaterplein 40, 3015 GD Rotterdam, The Netherlands; 8 Department of Radiology and Nuclear Medicine, Erasmus Medical Center, University Medical Center Rotterdam, Rotterdam, The Netherlands

**Keywords:** Right ventricle, ST-segment elevation myocardial infarction, Chronic total occlusion, Strain analysis, Cardiovascular magnetic resonance

## Abstract

The right ventricle (RV) is frequently involved in ST-segment elevation myocardial infarction (STEMI) when the culprit or concurrent chronic total occlusion (CTO) is located in the right coronary artery (RCA). We investigated RV function recovery in STEMI-patients with concurrent CTO. In EXPLORE, STEMI-patients with concurrent CTO were randomized to CTO percutaneous coronary intervention (PCI) or no CTO-PCI. We analyzed 174 EXPLORE patients with serial cardiovascular magnetic resonance imaging RV data (baseline and 4-month follow-up), divided into three groups: CTO-RCA (CTO in RCA, culprit in non-RCA; n = 89), IRA-RCA (infarct related artery [IRA] in RCA, CTO in non-RCA; n = 56), and no-RCA (culprit and CTO not in RCA; n = 29). Tricuspid annular plane systolic excursion (TAPSE), RV ejection fraction (RVEF), RV global longitudinal strain (GLS) and free wall longitudinal strain (FWLS) were measured. We found that RV strain and TAPSE improved in IRA-RCA and CTO-RCA (irrespective of CTO-PCI) at follow-up, but not in no-RCA. Only RV FWLS was different among groups at baseline, which was lower in IRA-RCA than no-RCA (− 26.0 ± 8.3% versus − 31.0 ± 6.4%, p = 0.006). Baseline RVEF, RV end-diastolic volume and TAPSE were associated with RVEF at 4 months. RV function parameters were not predictive of 4 year mortality, although RV GLS showed additional predictive value for New York Heart Association Classification > 1 at 4 months. In conclusion, RV parameters significantly improved in patients with acute or chronic RCA occlusion, but not in no-RCA patients. RV FWLS was the only RV parameter able to discriminate between acute ischemic and non-ischemic myocardium. Moreover, RV GLS was independently predictive for functional status.

**Supplementary Information:**

The online version contains supplementary material available at 10.1007/s10554-021-02423-9.

## Introduction

Assessment of the right ventricular (RV) function has prognostic implications in patients with ischemic heart disease [1]. The RV function can be expressed in global systolic function parameters, such as RV ejection fraction (RVEF) or tricuspid annular plane systolic excursion (TAPSE), but these parameters could overlook subtle and more regional dysfunction. A novel application of myocardial strain assessment using cardiovascular magnetic resonance imaging (CMR) derived myocardial feature-tracking (FT) enables advanced assessment of global and regional myocardial function [2]. Left ventricular strain analysis is able to accurately differentiate between infarcted and non-infarcted myocardium at segmental level [3]. Additionally, RV strain measurements are associated with global RV function and prognosis in patients with acute myocardial infarction [4–7].

In 15% of patients with an acute ST-segment elevation myocardial infarction (STEMI) a concomitant chronic total occlusion (CTO) is found—a 100% coronary lumen narrowing that is older than 3 months [8, 9]. In these patients, the RV function is frequently affected when the right coronary artery (RCA) is the infarct-related artery (IRA) or the CTO-related artery [10]. The difference in RV function and RV strain recovery between these patients with either acute or chronic ischemia of the RV, has never been described. Separately, it is assumed that RV dysfunction after STEMI may recover within a few months [11]. Moreover, RV dysfunction resulting from CTO RCA has also been thought to be able to improve [12]. Whether this is due to successful revascularization of CTO RCA, the development of a collateral network or to a temporal effect of hibernation has not yet been established.

The Evaluating Xience and left ventricular function in PCI on occlusiOns afteR STEMI (EXPLORE) trial randomized STEMI patients with a concurrent non-IRA CTO to additional CTO PCI or no CTO PCI after successful primary PCI [13]. We used the serial CMR data of EXPLORE trial participants who underwent CMR at baseline and at 4 months to evaluate the recovery of RV function parameters (global function as well as regional strain) from baseline to 4 month follow-up in patients with either an acute or chronic RCA occlusion, and to determine the prognostic value of the RV function parameters on prognosis. Furthermore, we aimed to determine the treatment effect of CTO PCI RCA on RV function recovery when compared to a control group of no CTO PCI.

## Methods

### Study design

The study design and outcomes of the EXPLORE trial have been published previously [13]. In short, 302 STEMI patients with concurrent CTO in non-IRA were randomly assigned to CTO PCI within 7 days after primary PCI (‘CTO PCI group’) or no-CTO PCI for at least 4 months (‘no-CTO PCI group’). To assess the primary endpoint of LV function, all patients underwent CMR at 4 months and preferably also at baseline. Clinical follow-up was obtained at 4 months and thereafter yearly until 5 years, and included functional status (angina and dyspnea) and survival status. Patients with factors hampering appropriate CMR, such as the presence of atrial fibrillation or cardiac devices at baseline, were excluded, as well as patients with hemodynamic instability > 48 h after STEMI. All inclusion and exclusion criteria have been published previously [14]. No significant difference was found for the primary endpoints of LVEF or LV end-diastolic volume between both treatment arms.

**Fig. 1:**
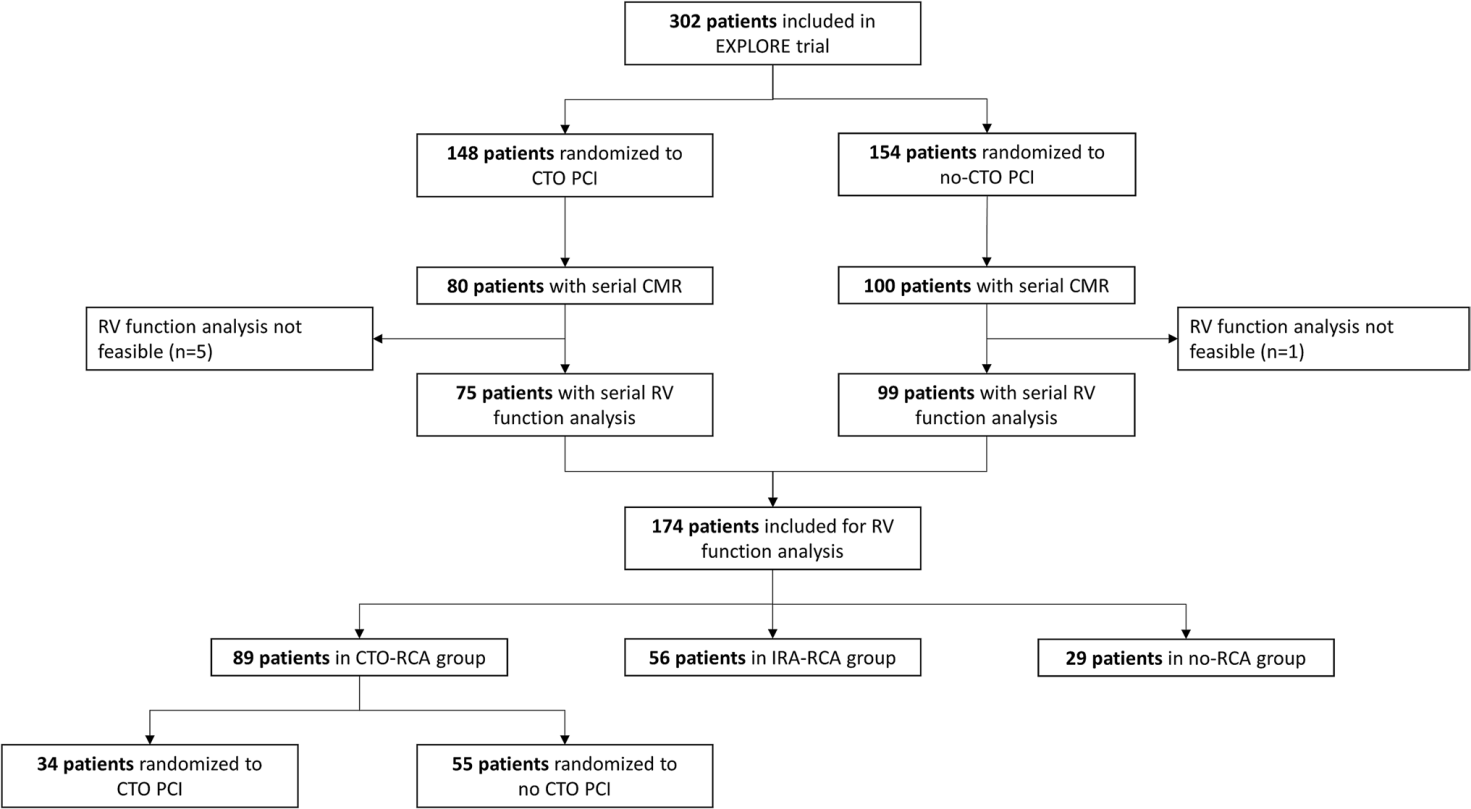
Flowchart of patient selection. *CMR* cardiovascular magnetic resonance; *CTO* chronic total occlusion; *IRA* infarct-related artery; *PCI* percutaneous coronary intervention; *RCA* right coronary artery; *RV* right ventricle

In the current substudy, we analyzed all patients who underwent baseline and follow-up CMR and excluded all patients without assessable RV images. The flowchart of patient selection is displayed in Fig. 1. A total of 180 patients underwent serial CMR imaging [15, 16]. In 6 of these patients, no RV analysis could be done at baseline or follow-up, the remaining 174 patients were included in this study. For the purpose of this study, we divided these 174 patients into three groups: (1) CTO-RCA (patients with CTO in RCA and culprit in non-RCA), (2) IRA-RCA (culprit in RCA and CTO in non-RCA), and (3) no-RCA (culprit and CTO in non-RCA). The CTO-RCA group consisted of patients that were randomly assigned to CTO PCI and patients that were assigned to no CTO PCI. All angiographies were evaluated by an independent angiography core lab. A CTO was defined as a 100% luminal narrowing without antegrade flow [8]. The collateral quality was scored using the Rentrop score [17]. Rentrop score 0 and 1 were considered as poorly-developed collaterals while Rentrop score 2 and 3 were considered as well-developed. The study endpoints were (1) the recovery of RV function parameters, i.e. RVEF, TAPSE, RV global longitudinal strain and RV free-wall longitudinal strain, from baseline to 4 months follow-up, (2) the impact of revascularization of the CTO on RV global function and RV strain recovery, and (3) the prognostic value of RV function and strain.

### Left ventricular CMR analysis

The imaging protocol and strain measurement technique have been published previously [15]. In short, electrocardiogram-gated steady-state free-precession cine images were obtained during repeated breath holds on 1.5-Tesla scanners. Late gadolinium enhanced images were used to identify the extent of the infarction. Left ventricular volumes have been measured previously for the primary outcome of the original EXPLORE study by an independent core laboratory, blinded for randomization outcome (ClinFact Corelab using Qmass MR analytical software version 7.6, Medis Medical Imaging Systems, Leiden, The Netherlands). Left ventricular myocardial longitudinal and circumferential strain measurement technique were described previously [15].

### Right ventricular CMR analysis

**Fig. 2:**
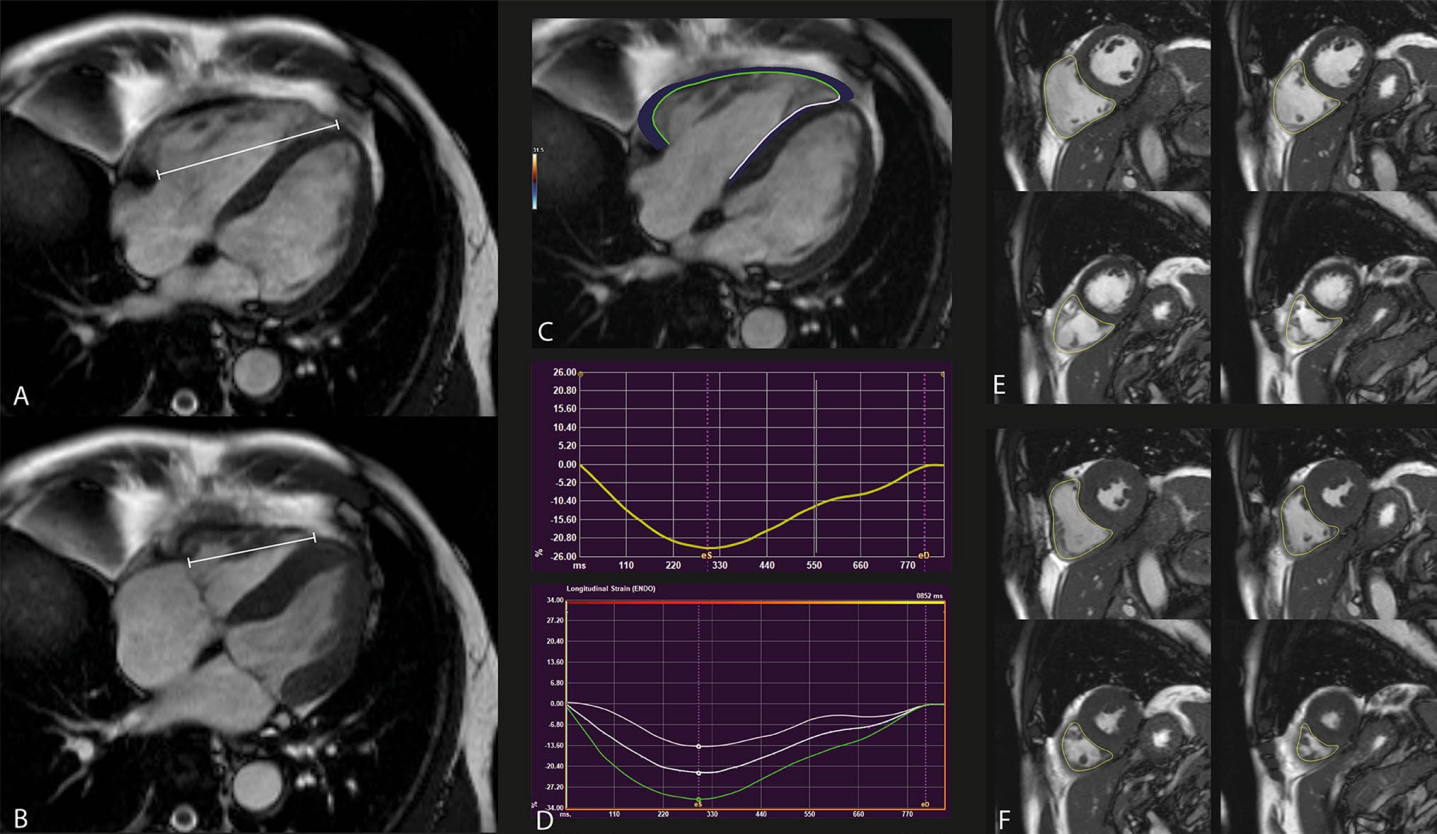
Measurement of right ventricular function parameters. **A** and **B** End-diastolic length (**A**) and end-systolic length (**B**) measurement for the calculation of the tricuspid annular plane systolic excursion; **C** Right ventricular strain measurement using feature tracking, with the green line indicating tracking of the free wall and white line indicating the septum tracking; **D** Strain curves, with the upper curve indicating the general RV strain curve and the lower curve indicating the RV strain curves divided into septum (white), average (white) and free wall (green). eS indicates end-systolic and eD indicates end-diastolic; **E**, **F** Right ventricular contours in the short-axis end-diastole (**E**) and end-systole (**F**) for RV volume measurements

Right ventricular myocardial strain was measured blinded for randomization outcome in the 4 chamber longitudinal axis images using commercially available FT-CMR software (QStrain, Medis Medical Imaging Systems version 2.0.12.2, Leiden, The Netherlands) under the supervision of an experienced CMR cardiologist (JE, supervision AH). The right ventricular endocardial contours were drawn manually during the end-diastolic and end-systolic phase. Subsequently, the software automatically traced the cardiac contours during the cardiac cycle, resulting in the peak global longitudinal strain (GLS) of the entire right ventricle, GLS of the RV free wall (FWLS) and GLS of the septum. Since deformation of the intraventricular septum is considered to be mainly affected by LV dysfunction, we chose to focus on the total RV GLS and the RV FWLS, rather than GLS of the septum [18]. RV volumes were measured in the short axis images and corrected for body-surface area (AvV, supervision AH). For the calculation of the TAPSE, we measured the RV length in the 4 chamber longitudinal axis from the apex to the juncture of the tricuspid annulus with the RV free wall in end-diastole (end-diastolic length; EDL) and end-systole (end-systolic length; ESL), as described previously [19]. Then, the TAPSE was calculated by subtracting the ESL from the EDL. An example of the measurement techniques is displayed in Fig. 2.

### Reproducibility analysis

We tested the intra- and interreproducibility of RV GLS, FWLS, TAPSE and RVEF measurements in 30 randomly selected scans. The measurements were repeated by the initial observer to measure the intraobserver variability and by a second observer to measure the interobserver variability, all blinded for the previous analyses.

### Statistical analysis

Statistical analyses were performed using SPSS Statistics version 25. Categorical data were described as frequencies with percentages and compared using the Fisher’s exact test to compare 2 groups or the Chi square test to compare > 2 groups. The normality of numerical data was assessed by evaluation of the histogram and in case of doubt using the Kolmogorov-Smirnoff test. Normally distributed numerical data were described as mean ± standard deviation (SD) and compared using the Student’s t-test for independent comparisons between 2 groups, the paired t-test for the comparison of 2 serial measurements within a group, and the one-way ANOVA for the independent comparison between 3 groups. When an overall significant ANOVA was found, post-hoc analyses were performed to identify subgroup differences. Pearson’s correlation coefficient analysis and scatter plots were used to study the correlation between the different RV function parameters. Multivariable linear regression was used to identify predictors for RVEF at 4 month follow-up and multivariable logistic regression was used to identify predictors for New York Heart Association (NYHA) Classification > 1 at 4 month follow-up. Using Cox proportional hazards models we tested the effect of RV GLS and RV FWLS on mortality and calculated hazard ratios (HR) with 95% confidence intervals (CI). Stepwise forward selection of variables was used for the regression models. Variables entered the models if p < 0.05 and were removed if p > 0.10. Inter- and intraobserver variability were calculated as intra-class correlations (ICC) using a model of absolute agreement. All tests were 2-sided and a p-value of < 0.05 was considered statistically significant.

## Results

### Baseline characteristics

The baseline characteristics of the included 174 patients are displayed in Table 1. The mean age was 60 ± 10 years and 87% was male (n = 151). Baseline CMR was performed at a median of 3 days (IQR 2–5) after primary PCI. Patients were divided into the three groups according to RCA involvement: (1) CTO-RCA (n = 89); 2) IRA-RCA (n = 56); 3) no-RCA (n = 29). There were no differences among the three groups regarding comorbidities. Patients in the CTO-RCA group had a higher Japan-CTO score (J-CTO) than patients in IRA-RCA group (2.4 ± 1.1 versus 1.8 ± 1.1; p = 0.004), more frequently had well-developed collaterals (69% versus 41% in the IRA-RCA group, p = 0.002) and a proximal location of the CTO (96% versus 35% in the no-RCA group, p < 0.001, and 66% in the IRA-RCA group, p < 0.001).

**Table 1:** Baseline characteristics

	Total	no-RCA	CTO-RCA	IRA-RCA	p-value^a^
	N = 174	N = 29	N = 89	N = 56	
Baseline characteristics					
Age, years	60 ± 10	61 ± 10	59 ± 10	60 ± 10	0.84
Male gender	151 (87)	27 (93)	79 (89)	45 (80)	0.19
Diabetes mellitus	27 (16)	6 (21)	14 (16)	7 (13)	0.61
Hypertension	76 (44)	12 (41)	42 (47)	22 (39)	0.62
Hypercholesterolemia	59 (34)	13 (45)	27 (30)	19 (34)	0.36
Current smoker	90 (52)	13 (45)	47 (53)	30 (54)	0.72
Previous myocardial infarction	25 (14)	7 (24)	15 (17)	3 (5)	0.041
Previous percutaneous coronary intervention	18 (10)	5 (17)	10 (11)	3 (5)	0.22
Familial history of coronary artery disease	70 (40)	11 (38)	37 (42)	22 (39)	0.93
Angiographic characteristics					
Multiple CTOs	20 (12)	2 (7)	14 (16)	4 (7)	0.20
Japan-CTO score	2.1 ± 1.1	2.0 ± 0.9	2.4 ± 1.1	1.8 ± 1.1	0.010
Poorly-developed collaterals to the CTO	75 (43)	14 (48)	28 (32)	33 (59)	0.004
Proximal location of CTO	132 (76)	10 (35)	85 (96)	37 (66)	< 0.001
IRA location					< 0.001
Left main/left anterior descending artery	75 (43)	20 (69)	55 (62)	–	
Left circumflex artery	43 (25)	9 (31)	34 (38)	–	
Right coronary artery	56 (32)	–	–	56 (100)	
Proximal location of IRA	135 (78)	20 (69)	61 (69)	54 (96)	< 0.001
Three-vessel disease	71 (41)	9 (31)	37 (42)	25 (45)	0.47
SYNTAX Score after Primary PCI	27 ± 9	30 ± 9	26 ± 9	27 ± 9	0.09
CMR characteristics					
Right ventricle					
End-diastolic volume, ml/m^2^	77 ± 20	73 ± 16	77 ± 20	78 ± 20	0.59
End-systolic volume, ml/m^2^	37 ± 12	35 ± 12	36 ± 12	38 ± 13	0.48
Ejection fraction, %	52 ± 9	53 ± 7	53 ± 9	51 ± 9	0.44
TAPSE, mm	19.2 ± 5.4	20.0 ± 5.7	19.6 ± 5.4	18.3 ± 5.2	0.26
Global longitudinal strain total, %	− 21.2 ± 6.0	− 22.9 ± 5.9	− 21.3 ± 5.9	− 20.2 ± 6.0	0.15
Global longitudinal strain free wall, %	− 28.0 ± 7.9	− 31.0 ± 6.4	− 28.3 ± 7.7	− 26.0 ± 8.3	0.018
Global longitudinal strain septum, %	− 14.4 ± 6.6	− 16.1 ± 6.6	− 13.7 ± 6.6	− 14.6 ± 6.5	0.24
Left ventricle					
End-diastolic volume, ml/m^2^	102 ± 23	102 ± 21	104 ± 22	97 ± 24	0.07
End-systolic volume, ml/m^2^	60 ± 22	62 ± 19	63 ± 24	55 ± 22	0.13
Ejection fraction, %	42 ± 11	40 ± 8	41 ± 13	43 ± 12	0.59
Global longitudinal strain, %	− 14.2 ± 6.4	− 10.7 ± 5.0	− 13.8 ± 6.3	− 16.5 ± 6.2	0.002
Global circumferential strain, %	− 19.7 ± 7.0	− 18.7 ± 5.4	− 18.9 ± 7.2	− 21.2 ± 7.2	0.24
Infarct size (culprit, MVO, CTO), g	11.8 ± 10.7	13.4 ± 12.5	13.3 ± 12.1	8.8 ± 5.7	0.07
Presence of MVO	60 (45)	13 (59)	31 (46)	16 (37)	0.24

Data are presented as numbers (percentages) or mean ± standard deviation

*CMR* cardiac magnetic resonance imaging; *CTO* chronic total occlusion; *IRA* infarct-related artery; *IQR* interquartile range; *MVO* microvascular obstruction; *RCA* right coronary artery; *SYNTAX Score* Synergy between PCI with Taxus and Cardiac Surgery Score; *TAPSE* tricuspid annular plane systolic excursion

^a^Outcomes were analyzed using the chi-square test for categorical data, the one-way ANOVA for normally distributed continuous data or the Kruskal–Wallis test for not-normally distributed continuous data

Baseline RV function was relatively preserved with a mean RVEF of 52% ± 9 and mean TAPSE of 19.2 mm ± 5.4. RVEF and TAPSE were not significantly different between the three groups. Baseline RV GLS was − 21.2 ± 6.0% and RV FWLS − 28.0% ± 7.9. The only RV parameter that was different among RCA groups at baseline was RV FWLS, which was significantly lower in the IRA-RCA group (− 26.0% ± 8.3) than in the no-RCA group (− 31.0% ± 6.4, p = 0.006).

### RV function recovery

**Fig. 3:**
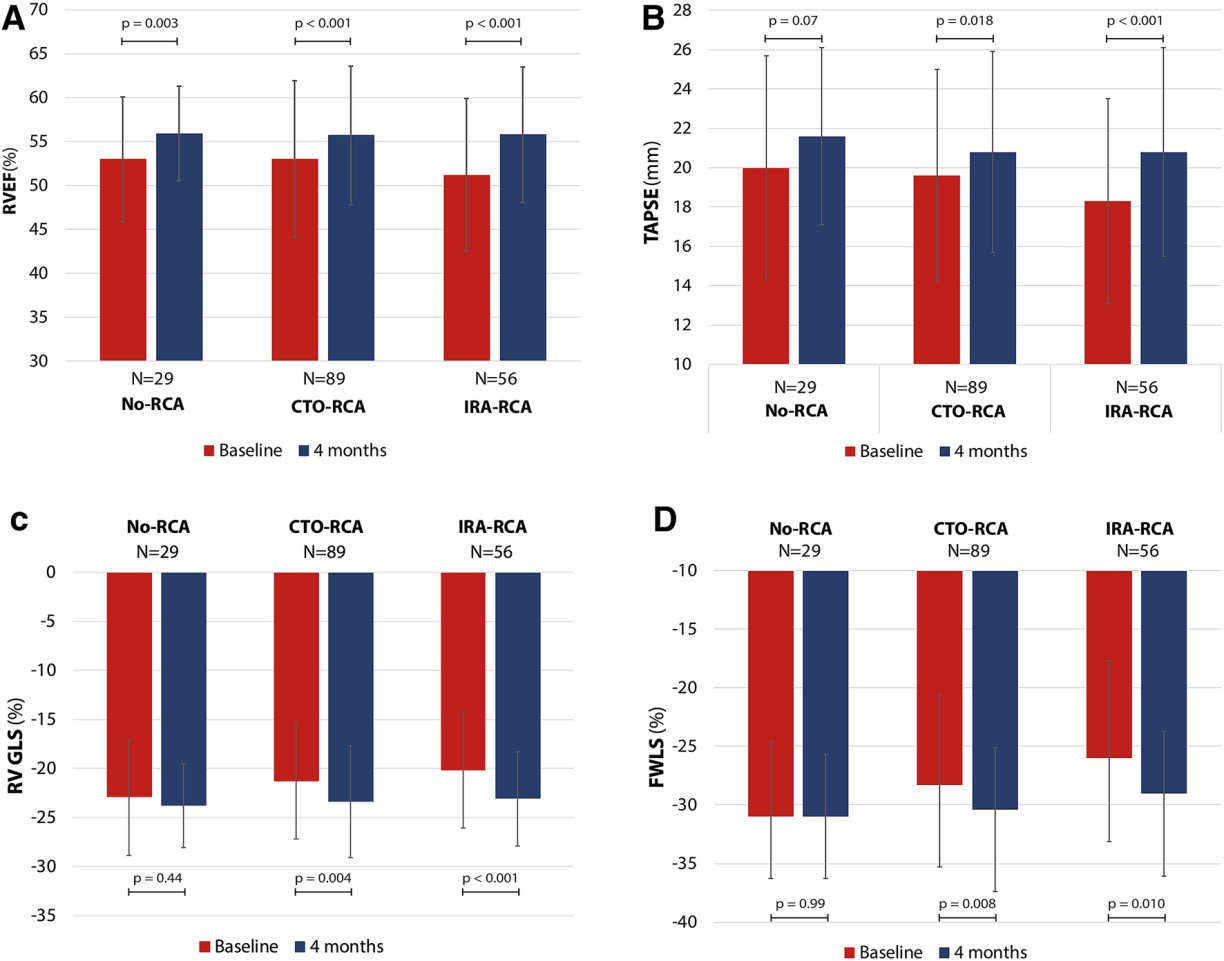
Recovery of different right ventricular function parameters. **A** RVEF recovery; **B** TAPSE recovery; **C** RV global longitudinal strain recovery; **D** RV free wall longitudinal strain recovery. *CTO* chronic total occlusion; *FWLS* free wall longitudinal strain; *IRA* infarct-related artery; *PCI* percutaneous coronary intervention; *RCA* right coronary artery; *RV* right ventricle; *RVEF* right ventricular ejection fraction; *TAPSE* tricuspid annular plane systolic excursion

Table 2 and Fig. 3 display the recovery of the different RV function parameters. RVEF improved significantly from baseline to 4 months follow-up in all groups. TAPSE improved only in the CTO-RCA (Δ1.3 mm ± 5.0, p = 0.018) and IRA-RCA groups (Δ2.5 mm ± 4.7, p < 0.001). The global RV GLS and RV FWLS improved significantly in the CTO-RCA group (Δ-2.1 ± 6.7, p = 0.004 and Δ-2.1 ± 7.2, p = 0.008) and the IRA-RCA group (Δ-2.8 ± 5.6, p < 0.001 and Δ-3.0 ± 8.4, p = 0.010), but not in the control group of no-RCA.

**Table 2:** Recovery of right ventricular function

		Total N = 174	No-RCA N = 29	CTO-RCA N = 89	IRA-RCA N = 56	p-value^a^
RVEF (%)	Baseline	52.4 ± 8.5	53.0 ± 7.1	53.0 ± 8.9	51.2 ± 8.7	0.44
	4 months	55.8 ± 7.5	55.9 ± 5.4	55.7 ± 7.9	55.8 ± 7.7	> 0.99
	Difference	3.4 ± 7.4	2.8 ± 6.2	2.8 ± 7.4	4.6 ± 8.0	0.32
	*p-value baseline vs 4 months*^*†*^	< 0.001	0.003	< 0.001	< 0.001	
TAPSE (mm)	Baseline	19.2 ± 5.4	20.0 ± 5.7	19.6 ± 5.4	18.3 ± 5.2	0.26
	4 months	20.9 ± 5.0	21.6 ± 4.5	20.8 ± 5.1	20.8 ± 5.3	0.73
	Difference	1.7 ± 4.8	1.6 ± 4.5	1.3 ± 5.0	2.5 ± 4.7	0.31
	*p-value baseline vs 4 months*^b^	< 0.001	0.07	0.018	< 0.001	
RV GLS (%)	Baseline	− 21.2 ± 6.0	− 22.9 ± 5.9	− 21.3 ± 5.9	− 20.2 ± 6.0	0.15
	4 months	− 23.4 ± 5.2	− 23.8 ± 4.3	− 23.4 ± 5.7	− 23.1 ± 4.8	0.82
	Difference	− 2.1 ± 6.3	− 0.9 ± 6.2	− 2.1 ± 6.7	− 2.8 ± 5.6	0.41
	*p-value baseline vs 4 months*^b^	< 0.001	0.44	0.004	< 0.001	
RV FWLS (%)	Baseline	− 28.0 ± 7.9	− 31.0 ± 6.4	− 28.3 ± 7.7	− 26.0 ± 8.3	0.018
	4 months	− 30.0 ± 6.8	− 31.0 ± 5.3	− 30.4 ± 7.0	− 29.0 ± 7.1	0.35
	Difference	− 2.0 ± 7.5	0.0 ± 6.6	− 2.1 ± 7.2	− 3.0 ± 8.4	0.22
	*p-value baseline vs 4 months*^b^	0.001	0.99	0.008	0.010	

*CTO* chronic total occlusion; FWLS = free wall longitudinal strain; *GLS* global longitudinal strain; *IRA* infarct-related artery; *RCA* right coronary artery; *RV* right ventricle; *RVEDV* right ventricular end-diastolic volume; *RVEF* right ventricular ejection fraction; *TAPSE* tricuspid annular plane systolic excursion

^a^Outcomes between the three RCA groups were compared using one-way ANOVA

^b^Outcomes between baseline and 4 months follow-up within groups was compared using the paired samples t-test

Within the total CTO-RCA group (n = 89), 34 patients were randomized to CTO PCI and 55 patients were randomized to no CTO PCI. Among these 34 patients the procedural success rate was 67.6% (n = 23/34). The recovery of the RV function parameters RVEF, TAPSE and RV FWLS was comparable among randomization groups (Supplemental Table 1). In the CTO-RCA group, PCI of the CTO did not lead to improvement of the RV GLS or RV FWLS from baseline to follow-up.

### Correlation of RV function parameters

Correlation between the three RV function parameters RVEF, TAPSE and RV FWLS was calculated. All combinations showed significant correlations. The scatterplots are displayed in Supplemental Fig. 1. The strongest correlation was found between TAPSE and RV FWLS (Pearson’s *r* =  − 0.56, p < 0.001).

### The prognostic value of RV function parameters

In univariable linear regression, male gender, as well as all baseline RV function parameters, including global RV GLS and RV FWLS, were significant predictors for RVEF at 4-month follow-up. In stepwise forward selection of variables, baseline RVEF, baseline RVEDV and baseline TAPSE were independent predictors (Table 3).

**Table 3:** Multivariable linear regression model for the prediction of right ventricular ejection fraction at 4 months follow-up

Univariable linear regression					Multivariable linear regression				
	Beta	95% confidence interval		p-value^a^		Beta	95% confidence interval		p-value*
		Lower limit	Upper limit				Lower limit	Upper limit	
Age (years)	0.07	− 0.04	0.18	0.23					
Male gender	− 3.33	− 6.60	− 0.06	0.046					
Diabetes	1.03	− 2.06	4.12	0.51					
CTO in RCA	0.29	− 1.96	2.53	0.80					
IRA in RCA	0.03	− 2.36	2.43	0.98					
Randomisation to CTO PCI	− 1.20	− 3.45	1.06	0.30					
Baseline RVEF (%)	0.51	0.40	0.61	< 0.001	Baseline RVEF (%)	0.43	0.32	0.55	< 0.001
Baseline RVEDV (ml/m^2^)	− 0.11	− 0.16	− 0.05	< 0.001	Baseline RVEDV (ml/m^2^)	− 0.11	− 0.16	− 0.06	< 0.001
Baseline RV GLS (%)	− 0.35	− 0.53	− 0.17	< 0.001					
Baseline RV FWLS (%)	− 0.31	− 0.44	− 0.17	< 0.001					
Baseline TAPSE (mm)	0.35	0.15	0.55	0.001	Baseline TAPSE (mm)	0.20	0.01	0.39	0.044

*CTO* chronic total occlusion; *IRA* infarct-related artery; *RCA* right coronary artery; *RV* right ventricle; *RVEDV* right ventricular end-diastolic volume; *RVEF* right ventricular ejection fraction; *TAPSE* tricuspid annular plane systolic excursion

^a^A stepwise forward selection of variables was used for multivariable linear regression

In total, 8 of the 174 patients died (4.6%) during a median follow-up of 4 years (IQR 2–5). The mortality rate was comparable between groups (1/29 no-RCA patients, 5/89 CTO-RCA patients and 2/56 IRA-RCA patients, p = 0.81). A multivariate cox proportional hazards model was created to test baseline and CMR characteristics as predictors for mortality (Supplemental Table 2). With a stepwise forward selection of variables, both age (HR 1.12 per year increment, 95% CI 1.02–1.24, p = 0.030) and baseline LV GCS (HR 1.18, 95% CI 1.02–1.35, p = 0.022) remained significant predictors. We tested the impact of RV CMR variables the clinical outcome NYHA classification. A total of 75.3% (n = 131) had NYHA I, 19.5% (n = 34) had NYHA II and 2.9% (n = 5) had NYHA III at 4 month follow-up. In multivariable logistic regression, both RV GLS (OR 1.11, 95% CI 1.01–1.21, p = 0.024) and LVEF (OR 0.94, 95% CI 0.91–0.98, p = 0.002) were significantly associated with NYHA class II-III (Supplemental Table 3).

### Reproducibility

The reproducibility of the RV parameters is demonstrated in Supplemental Table 4. The inter- and intraobserver variability was 0.66 (95% CI 0.01–0.88) and 0.73 (95% CI 0.51–0.86) respectively for RVEF, and 0.80 (95% CI 0.46–0.92) and 0.93 (95% CI 0.86–0.97) for TAPSE. The RV strain measurements showed an excellent intraobserver variability (ICC for RV FWLS 0.89, 95% CI 0.77–0.95; and ICC for RV GLS 0.83, 95% CI 0.68–0.92) and interobserver variability for RV GLS (ICC 0.78, 95% CI 0.58–0.89), but a modest interobserver variability for RV FWLS (ICC 0.61, 95% 0.19–0.82).

## Discussion

In this CMR substudy of the EXPLORE trial, we evaluated the recovery and clinical value of RV function and RV strain parameters in patients with acute, chronic or no occlusion of the right coronary artery. We demonstrated that (1) RV free wall longitudinal strain significantly differed at baseline between the three groups with RV FWLS being the lowest in the patients with IRA-RCA compared to no-RCA, (2) RV strain, RV ejection fraction and TAPSE significantly improved from baseline to 4 months follow-up in patients with an acute infarction in the RCA and in patients with a CTO in the RCA (irrespective of revascularization of the CTO), and (3) RV global longitudinal strain was an independent predictor for functional dyspnea status at follow-up. However, RV strain was not predictive for RV ejection fraction or mortality in our cohort.

### RV strain

Disruption of the coronary blood flow of more than 30 min, in the setting of acute myocardial infarction, results in reduction of the myocardial contractility [20]. When the coronary hypoperfusion is transient due to restoration of the blood flow, this contractility may recover completely, although it can persist for several days-weeks after revascularization. This initial contractile dysfunction in the setting of restored blood flow is called “stunning”, which is a defense mechanism of the cardiomyocytes to preserve the cardiac function in case of hypoxia, by downregulating the glucose uptake and oxidative metabolism [21]. Furthermore, contractile dysfunction could also exist in the setting of reduced coronary blood flow to the myocardium, which is called “hibernation”, resulting from chronic hypoperfusion or after repetitive stunning [22]. Stunning and hibernation may occur locally in the myocardium subtended by the occluded artery, which in result, does not necessarily lead to impaired global function of the ventricle. This subclinical, regional dysfunction may be visualized with strain analysis. This technique allows the detection of minimal subtle myocardial deformation, that does not yet translate into global ventricular dysfunction. The tracing of myocardial borders to obtain strain measurements can be performed in echocardiography by speckle-tracking [23]. Feature-tracking strain measurement by CMR shows significant correlations to this previously established imaging technique, however, several studies imply that the modalities should not be used interchangeably but as substitute to the other [24, 25]. The reproducibility of RV strain measurements showed good results for the intra-observer measurements (ICC 0.89 and 0.83), but was limited for inter-observer measurements (ICC 0.61 for RV FWLS). These numbers are comparable with previously described reproducibility rates for RV strain measured in Medis Medical Imaging software: intra-observer varying between 0.88 and 0.99 and inter-observer variability between 0.58 and 1.00 [25–27]. Additionally, RV strain measurements are susceptible to effects of training [26].

Indeed, we found that RV FWLS was significantly more impaired at baseline in the IRA-RCA group, compared to patients with no RCA involvement, while none of the global RV function parameters showed a significant difference between the three groups. This finding illustrates the additional value of RV strain measurement in the assessment of RV function, since it might detect early local deformation before the global ventricular function is affected. An alternative more rapid measurement technique for RV deformation is RV long-axis strain, which could be a complementary parameter for a complete RV functional assessment [28].

### RV function recovery

The RV function (RVEF and TAPSE) and RV strain (RV GLS and RV FWLS) improved significantly from baseline to follow-up in patients in the IRA-RCA group and in patients in the CTO-RCA group (regardless of CTO revascularization). While the baseline LVEF was impaired in our study, the RV function parameters appeared to be within normal range at both baseline and follow-up. It should be noted that the preserved RV function parameters at baseline could have led to a reduced ability to find an impact of treatment on RV function recovery. Nonetheless, an improvement of the RV parameters was still observed. An improvement of RVEF was even observed in the no-RCA group, while none of the other RV parameters (TAPSE and RV strain) improved. We hypothesize that, although our study was limited by a low number of patients, the RV global function could be more congruent with the LV function and less affected by local deformation.

In patients with CTO-RCA, a previous observational echocardiography study found that the RV function (volumetric and strain) significantly improved 1 month after successful PCI of a CTO in the RCA, compared to baseline [12]. Contrarily, in our prospective randomized study, we found that RV function significantly improved in patients with a CTO-RCA, but this was irrespective of treatment allocation. It has been assumed that the vulnerability of the RV for persistent ischemic damage, in contrast to the LV, is lower [29]. This is a result of a lower oxygen demand by the RV due to the thinner wall and lower afterload, and a higher potential for collateral circulation [30, 31]. Therefore, we postulate that a CTO in the RCA less frequently leads to persistent impairment of RV function, in contrast to the impact of a left-sided CTO on LV function. We demonstrated that the collateral network, associated with conservation of cardiac function [32], was better developed in patients in the CTO-RCA group compared to the other groups (with a CTO in the left anterior descending artery or circumflex artery). Hence, in these patients with a well-developed collateral circulation and preserved RV function, the additional effect of revascularization of a CTO on myocardial contractility is probably limited.

### Clinical implications

In contrast to previous findings by Lu et al. [6], in our study baseline RV strain did not appear to be an independent predictor for RVEF at follow-up. Moreover, in speckle-tracking echocardiography studies by Park et al. [7] and Gavazzoni et al. [33], RV longitudinal strain was predictive for mortality and major adverse cardiovascular events or hospital admissions for heart failure. However, in our current CMR study, we found that neither RVEF nor RV strain were predictors for mortality, but our event numbers were low. It should be noted that the agreement of the different strain imaging modalities, using echocardiography as well as CMR, is limited [25], thus the modalities should probably not be used interchangeably.

As was suggested by several studies, RV strain may be used to predict functional outcome in patients with several etiologies of heart failure [7, 33–35]. To determine the prognostic value of RV function and strain on heart failure and functional outcome, we used NYHA class as a surrogate. A previous study described a correlation between RV strain and NYHA class [36]. In addition, we found that RV strain was independently associated with reduced functional status at 4 month follow-up. However, we could not confirm the previously described association with mortality [4, 7]. This could partially be explained by the fact that this is a sub-analysis of the EXPLORE trial, which aimed to find differences after randomization to CTO PCI or no CTO PCI. Accordingly, the predefined study endpoints of the EXPLORE trial are mostly coronary events.

Strain analysis is an evolving technique that allows a quick and effortless evaluation of local deformation. RV strain showed significant correlation with the previously established RV functional parameters, RVEF and TAPSE, and could therefore also be of predictive value for clinical outcome, although this could not be demonstrated in our study due to low event numbers. Additionally, we found that strain was the only determinant that was able to discriminate between acute ischemic and non-ischemic myocardium, thus possibly has the ability to detect early local myocardial dysfunction. Strain analysis should probably not be considered a substitute for RVEF, but as an additional parameter for more complete RV function assessment.

### Limitations

Some limitations should be acknowledged. First, we aimed to divide patients into groups that illustrated the RV involvement by IRA or CTO. However, by discriminating based on the presence of RCA disease, variations in coronary anatomy are not completely taken into account, since occlusions in the left anterior descending artery or the left circumflex artery may also lead to some RV involvement. Second, we tried to explore the predictive value of RV function on clinical follow-up, however, our sample size and event rates are too small to draw definite conclusions. Therefore, these results should be interpreted with caution and should be tested in a larger long-term cohort. The studied population had relatively preserved RV function thus strong associations on RV deformation would be hard to find. Third, the inter- and intraobserver reproducibility of the RV parameters is limited and potentially influenced by software type and no standards for FT-strain exist to date.

## Conclusion

In this RV CMR substudy of the EXPLORE trial, we demonstrated that RV strain, RVEF and TAPSE improved significantly from baseline to 4 months follow-up in patients with acute or chronic occlusion in the RCA. In an exploratory analysis, no treatment effect of PCI CTO of the RCA was found on RV function recovery. RV free wall longitudinal strain was, however, able to discriminate between acute ischemic and non-ischemic myocardium. RV strain did not have an additional predictive value on mortality, although limited by a low number of events. Still, RV strain was an additional predictor for functional status in terms of NYHA classification at 4 months follow-up.

## Supplementary Information

Below is the link to the electronic supplementary material.

Supplementary file 1 (DOCX 639 kb)

## Data Availability

Upon request.
