# Supplementary material for: Recovery of right ventricular function and strain in patients with ST-segment elevation myocardial infarction and concurrent chronic total occlusion
Source: Int J Cardiovasc Imaging. 2021 Sep 23;38(3):631–41. doi: 10.1007/s10554-021-02423-9 (PMC8926979; doi:10.1007/s10554-021-02423-9)
Supplement: Supplementary file 1 — (DOCX 639 kb) [file 10554_2021_2423_MOESM1_ESM.docx]

**SUPPLEMENTAL MATERIALS**

1. **Supplemental Table 1.** Recovery of right ventricular function in CTO-RCA group, stratified for randomization group.
2. **Supplemental Table 2.** Multivariable Cox proportional hazard model for the prediction of mortality until 5 years follow-up.
3. **Supplemental Table 3.** Multivariable logistic regression model for the association with New York Heart Association classification II-III at 4 months follow-up.
4. **Supplemental Table 4.** Reproducibility rates for right ventricular parameters.
5. **Supplemental Figure 1.** Correlations between different right ventricular function parameters.

|  |  | **CTO-RCA**  N=89 | **CTO PCI**  N=34 | **no CTO PCI**  N=55 | *p-value  PCI vs no PCI** | |
| --- | --- | --- | --- | --- | --- | --- |
| **RVEF** (%) | Baseline | 53.0 ±8.9 | 51.9 ±8.4 | 53.6 ±9.2 | 0.39 |  |
|  | 4 months | 55.7 ±7.9 | 54.5 ±8.2 | 56.5 ±7.8 | 0.27 |  |
|  | Difference | 2.8 ±7.4 | 2.6 ±6.4 | 2.8 ±8.0 | 0.89 |  |
|  | *p-value baseline vs 4 months^†^* | <0.001 | 0.024 | 0.011 |  |  |
| **TAPSE** (mm) | Baseline | 19.6 ±5.4 | 18.0 ±5.1 | 20.5 ±5.4 | 0.027 |  |
|  | 4 months | 20.8 ±5.1 | 19.8 ±5.5 | 21.5 ±4.7 | 0.14 |  |
|  | Difference | 1.3 ±5.0 | 1.9 ±5.4 | 0.9 ±4.7 | 0.39 |  |
|  | *p-value baseline vs 4 months^†^* | 0.018 | 0.052 | 0.16 |  |  |
| **RV GLS** (%) | Baseline | -21.3 ±5.9 | -21.2 ±4.6 | -21.4 ±6.7 | 0.83 |  |
|  | 4 months | -23.4 ±5.7 | -22.0 ±5.8 | -24.3 ±5.5 | 0.06 |  |
|  | Difference | -2.1 ±6.7 | -0.8 ±5.4 | -2.9 ±7.3 | 0.16 |  |
|  | *p-value baseline vs 4 months^†^* | 0.004 | 0.39 | 0.005 |  |  |
| **RV FWLS** (%) | Baseline | -28.3 ±7.7 | -28.8 ±6.9 | -28.1 ±8.2 | 0.69 |  |
|  | 4 months | -30.4 ±7.0 | -29.6 ±8.0 | -30.9 ±6.3 | 0.41 |  |
|  | Difference | -2.1 ±7.2 | -0.9 ±7.0 | -2.8 ±7.2 | 0.22 |  |
|  | *p-value baseline vs 4 months^†^* | 0.008 | 0.48 | 0.006 |  |  |

**Supplemental Table 1.** Recovery of right ventricular function in CTO-RCA group, stratified for randomization group.

CTO = chronic total occlusion; FWLS = free wall longitudinal strain; GLS = global longitudinal strain; IRA = infarct-related artery; PCI = percutaneous coronary intervention; RCA = right coronary artery; RV = right ventricle; RVEDV = right ventricular end-diastolic volume; RVEF = right ventricular ejection fraction; TAPSE = tricuspid annular plane systolic excursion. * Outcomes between randomization groups in the CTO-RCA group were compared using the independent samples t-test. † Outcomes between baseline and 4 months follow-up within groups was compared using the paired samples t-test.

**Supplemental Table 2.** Multivariable Cox proportional hazard model for the prediction of mortality until 5 years follow-up.

| **Univariable Cox regression** | | | | | **Multivariable Cox regression** | | | | |
| --- | --- | --- | --- | --- | --- | --- | --- | --- | --- |
|  |  | 95% confidence interval | |  |  |  | 95% confidence interval | |  |
|  | HR | Lower limit | Upper limit | p-value* |  | HR | Lower limit | Upper limit | p-value* |
| **Age** (years) | 1.12 | 1.02 | 1.22 | 0.014 | **Age** (years) | 1.12 | 1.02 | 1.24 | 0.030 |
| **Male gender** | 1.01 | 0.12 | 8.19 | >0.99 |  |  |  |  |  |
| **Diabetes** | 0.49 | 0.10 | 2.42 | 0.49 |  |  |  |  |  |
| **CTO in RCA** | 1.17 | 0.29 | 4.70 | 0.82 |  |  |  |  |  |
| **IRA in RCA** | 0.62 | 0.13 | 3.10 | 0.56 |  |  |  |  |  |
| **Randomisation to CTO PCI** | 2.13 | 0.51 | 8.92 | 0.30 |  |  |  |  |  |
| **Baseline RVEF** (%) | 0.94 | 0.87 | 1.02 | 0.14 |  |  |  |  |  |
| **Baseline RVEDV** (ml/m^2^) | 0.99 | 0.95 | 1.03 | 0.57 |  |  |  |  |  |
| **Baseline RV GLS** (%) | 1.06 | 0.94 | 1.21 | 0.35 |  |  |  |  |  |
| **Baseline RV FWLS** (%) | 1.00 | 0.92 | 1.09 | 0.96 |  |  |  |  |  |
| **Baseline TAPSE** (mm) | 1.04 | 0.91 | 1.19 | 0.62 |  |  |  |  |  |
| **Baseline LVEF** (%) | 0.93 | 0.88 | 0.99 | 0.020 |  |  |  |  |  |
| **Baseline LVDEDV** (ml/m^2^) | 1.03 | 1.00 | 1.05 | 0.019 |  |  |  |  |  |
| **Baseline LV GLS** (%) | 1.07 | 0.95 | 1.21 | 0.29 |  |  |  |  |  |
| **Baseline LV GCS** (%) | 1.15 | 1.02 | 1.30 | 0.028 | **Baseline LV GCS** (%) | 1.18 | 1.02 | 1.35 | 0.022 |

CTO = chronic total occlusion; GCS = global circumferential strain; GLS = global longitudinal strain; HR = hazard ratio. IRA = infarct-related artery; LV = left ventricle; LVEDV = left ventricular end-diastolic volume; LVEF = left ventricular ejection fraction; RCA = right coronary artery; RV = right ventricle; RVEDV = right ventricular end-diastolic volume; RVEF = right ventricular ejection fraction; TAPSE = tricuspid annular plane systolic excursion. *A stepwise forward selection of variables was used for multivariable Cox regression.

**Supplemental Table 3.** Multivariable logistic regression model for the association with New York Heart Association classification II-III at 4 months follow-up.

| **Univariable logistic regression** | | | | | **Multivariable logistic regression** | | | | |
| --- | --- | --- | --- | --- | --- | --- | --- | --- | --- |
|  |  | 95% confidence interval | |  |  |  | 95% confidence interval | |  |
|  | OR | Lower limit | Upper limit | p-value* |  | OR | Lower limit | Upper limit | p-value* |
| **Age** (years) | 1.01 | 0.98 | 1.05 | 0.48 |  |  |  |  |  |
| **Male gender** | 1.01 | 0.35 | 2.95 | 0.98 |  |  |  |  |  |
| **Diabetes** | 0.50 | 0.20 | 1.23 | 0.13 |  |  |  |  |  |
| **CTO in RCA** | 0.87 | 0.43 | 1.78 | 0.70 |  |  |  |  |  |
| **IRA in RCA** | 1.27 | 0.60 | 2.70 | 0.53 |  |  |  |  |  |
| **Randomisation to CTO PCI** | 0.57 | 0.27 | 1.21 | 0.15 |  |  |  |  |  |
| **Baseline RVEF** (%) | 0.93 | 0.89 | 0.98 | 0.002 |  |  |  |  |  |
| **Baseline RVEDV** (ml/m^2^) | 0.98 | 0.96 | 1.00 | 0.032 |  |  |  |  |  |
| **Baseline RV GLS** (%) | 1.10 | 1.03 | 1.17 | 0.007 | **Baseline RV GLS** (%) | 1.11 | 1.01 | 1.21 | 0.024 |
| **Baseline RV FWLS** (%) | 1.05 | 1.00 | 1.10 | 0.06 |  |  |  |  |  |
| **Baseline TAPSE** (mm) | 0.88 | 0.82 | 0.95 | 0.001 |  |  |  |  |  |
| **Baseline LVEF** (%) | 0.93 | 0.90 | 0.96 | <0.001 | **Baseline LVEF** (%) | 0.94 | 0.91 | 0.98 | 0.002 |
| **Baseline LVDEDV** (ml/m^2^) | 1.02 | 1.00 | 1.03 | 0.018 |  |  |  |  |  |
| **Baseline LV GLS** (%) | 1.10 | 1.04 | 1.17 | 0.002 |  |  |  |  |  |
| **Baseline LV GCS** (%) | 1.12 | 1.05 | 1.19 | 0.001 |  |  |  |  |  |

CTO = chronic total occlusion; GCS = global circumferential strain; GLS = global longitudinal strain; IRA = infarct-related artery; LV = left ventricle; LVEDV = left ventricular end-diastolic volume; LVEF = left ventricular ejection fraction; OR = odds ratio; RCA = right coronary artery; RV = right ventricle; RVEDV = right ventricular end-diastolic volume; RVEF = right ventricular ejection fraction; TAPSE = tricuspid annular plane systolic excursion. *A stepwise forward selection of variables was used for multivariable logistic regression.

|  | **Intra-observer** | **Inter-observer** |
| --- | --- | --- |
|  | **ICC (95% CI)** | **ICC (95% CI)** |
| **RVEF** | 0.73 (0.51 – 0.86) | 0.66 (0.01 – 0.88) |
| **TAPSE** | 0.93 (0.86 – 0.97) | 0.80 (0.46 – 0.92) |
| **RV GLS** | 0.83 (0.68 – 0.92) | 0.78 (0.58 – 0.89) |
| **RV FWLS** | 0.89 (0.77 – 0.95) | 0.61 (0.19 – 0.82) |

**Supplemental Table 4.** Reproducibility rates for right ventricular parameters.

Measurements were performed in 30 randomly selected scans. Intra-class correlation coefficients were calculated using a model of absolute agreement.

FWLS = free-wall longitudinal strain; ICC = intra-class correlation; RV = right ventricle; RVEF = right ventricular ejection fraction; TAPSE = tricuspid annular plane systolic excursion.

**Supplemental Figure 1.** Correlations between different right ventricular function parameters.

**(A**) Scatterplot of right ventricular free wall longitudinal strain and TAPSE*;*

*
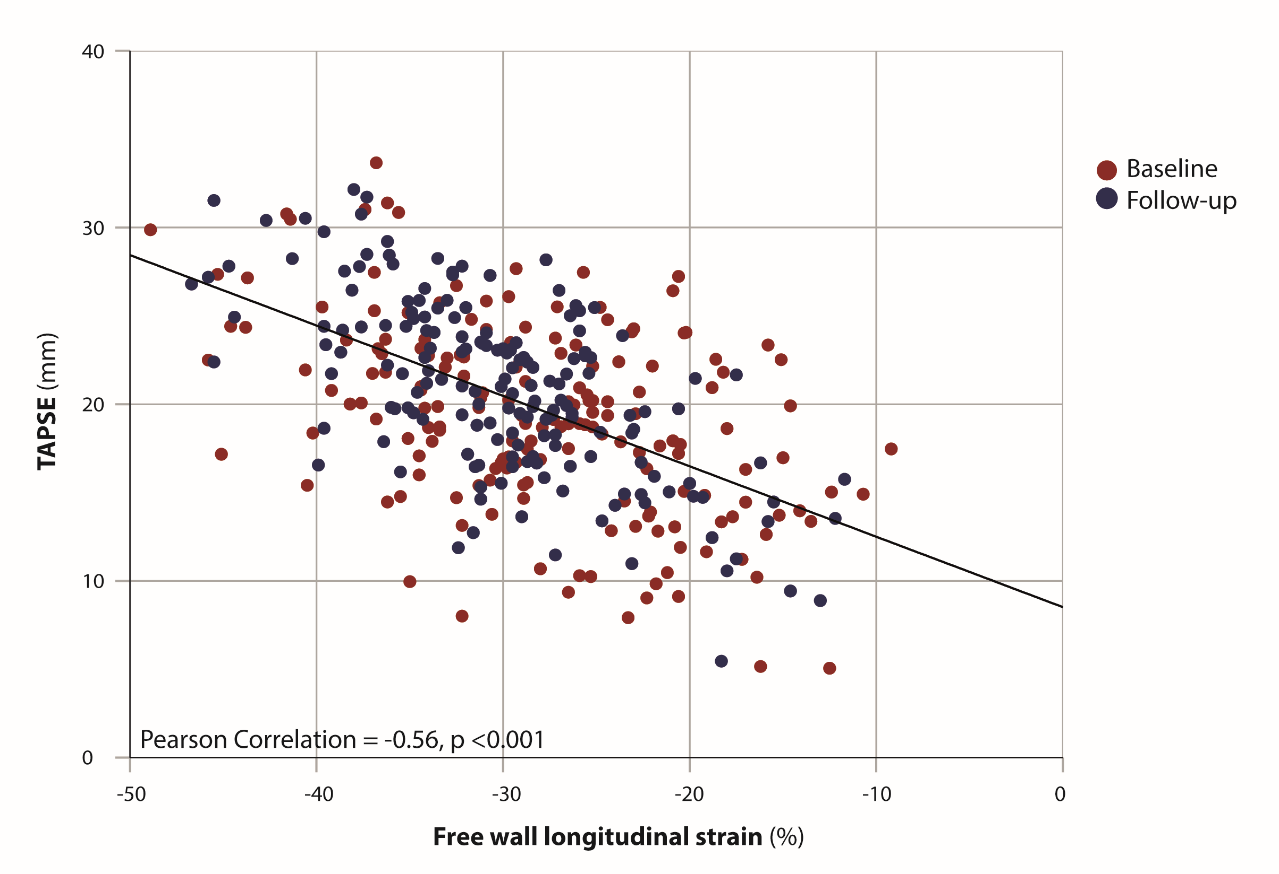
*

**(B)** Scatterplot of right ventricular ejection fraction and TAPSE;


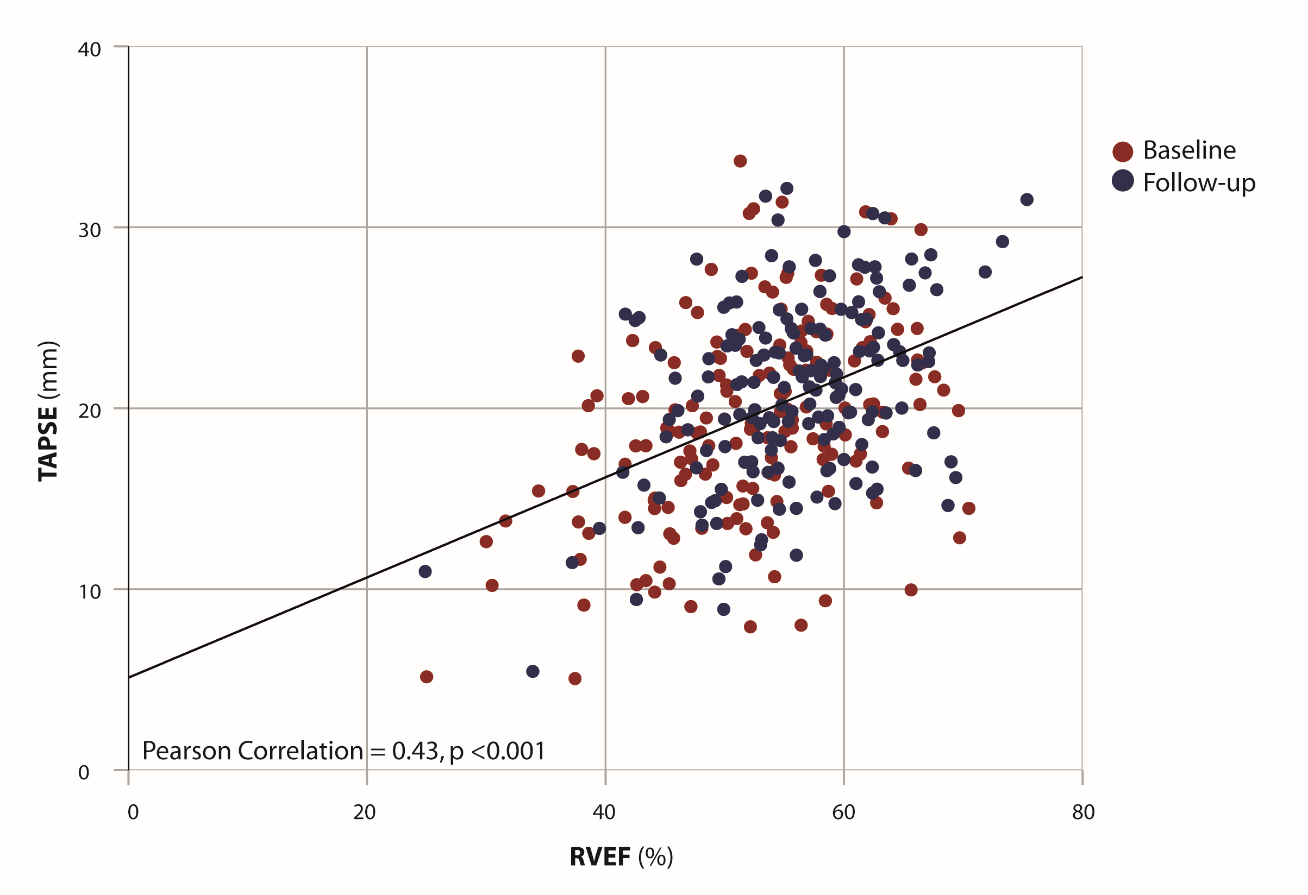


**(C)** Scatterplot of right ventricular ejection fraction and right ventricular free wall longitudinal strain.


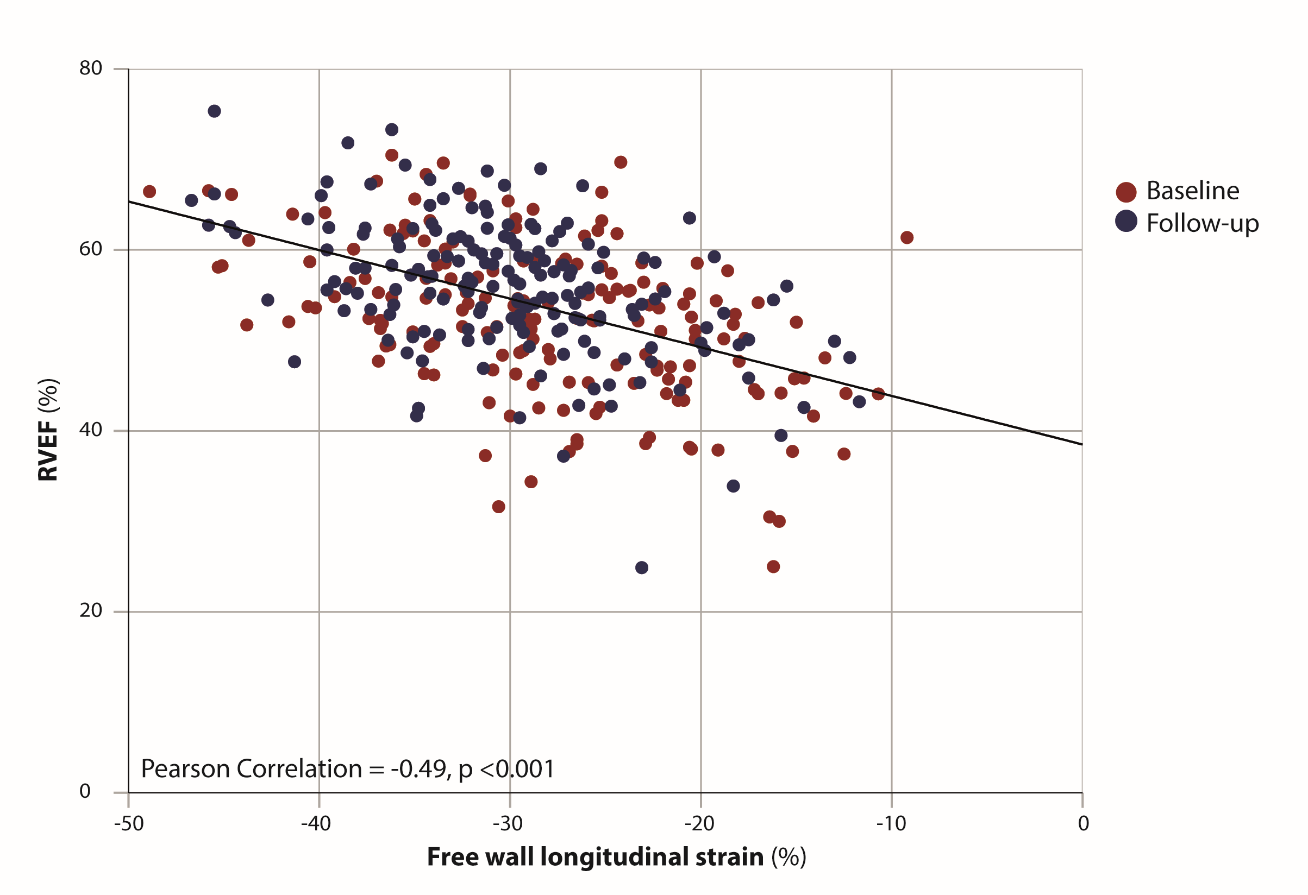


RVEF = right ventricular ejection fraction; TAPSE = tricuspid annular plane systolic excursion.
